# Community participation and private sector engagement are fundamental to achieving universal health coverage and health security in Africa: reflections from the second Africa health forum

**DOI:** 10.1186/s12919-019-0170-0

**Published:** 2019-11-12

**Authors:** Olushayo Olu, Pamela Drameh-Avognon, Emil Asamoah-Odei, Francis Kasolo, Thomas Valdez, Grace Kabaniha, Humphrey Karamagi, Suvajee Good, Helena O’Malley, Zabulon Yoti, Nirina Razakazoa, Etienne Minkoulou, Jean-Marie Dangou, Symplice Mbola Mbassi, Mariano Salazar Castellon, Joseph Cabore, Matshidiso Moeti

**Affiliations:** 1 WHO Country Office, Juba, Republic of South Sudan; 2 WHO Regional Office for Africa, Brazzaville, Republic of the Congo; 3 Consultant, Regional Director’s Office, WHO Regional Office for Africa, Brazzaville, Republic of the Congo; 4 WHO Country Office, Praia, Cabo Verde

**Keywords:** Second WHO Africa health forum, Community engagement and participation, Private sector engagement, Multisectoral collaboration, Health in all policies, Sustainable development goals, Universal health coverage, Health security, Africa

## Abstract

**Background:**

Inadequate access to quality health care services due to weak health systems and recurrent public health emergencies are impediments to the attainment of Universal Health Coverage and health security in Africa. To discuss these challenges and deliberate on plausible solutions, the World Health Organization Regional Office for Africa, in collaboration with the Government of Cabo Verde, convened the second Africa Health Forum in Praia, Cabo Verde on 26–28 March 2019, under the theme Achieving Universal Health Coverage and Health Security: The Africa We Want to See.

**Methods:**

The Forum was conducted through technical sessions consisting of high-level, moderated panel discussions on specific themes, some of them preceded by keynote addresses. There were booth exhibitions by Member States, World Health Organization and other organizations to facilitate information exchanges. A Communiqué highlighting the recommendations of the Forum was issued during the closing ceremony***.*** More than 750 participants attended. Relevant information from the report of the Forum and notes by the authors were extracted and synthesized into these proceedings.

**Conclusions:**

The Forum participants agreed that the role of community engagement and participation in the attainment of Universal Health Coverage, health security and ultimately the Sustainable Development Goals cannot be overemphasized. The public sector of Africa alone cannot achieve these three interrelated goals; other partners, such as the private sector, must be engaged. Technological innovations will be a key driver of the attainment of these goals; hence, there is need to harness the comparative advantages that they offer. Attainment of the three goals is also intertwined – achieving one paves the way for achieving the others. Thus, there is need for integrated public health approaches in the planning and implementation of interventions aimed at achieving them.

**Recommendations:**

To ensure that the recommendations of this Forum are translated into concrete actions in a sustainable manner, we call on African Ministers of Health to ensure their integration into national health sector policies and strategic documents and to provide the necessary leadership required for their implementation. We also call on partners to mainstream these recommendations into their ongoing support to World Health Organization African Member States.

## Background

The public health situation in Africa has continued to improve in the past few years, although the improvements are minor in comparison to what has been achieved in other parts of the world. For instance, while the healthy life expectancy (a measure of life expectancy adjusted for years spent with disability) increased from 50.9 years in 2012 to 53.8 years in 2015 [1], it is very low when compared with the global average of 72 years [2]. This trend is largely due to the inability of African populations to access affordable and quality health care, which is due to the lack of health care workers, weak supply chain systems for medical products, inadequate health financing and weak health governance [3].

The dire public health situation in Africa is further compounded by perennial public health emergencies, which often decimate the already-weak health care delivery system. The region experiences more than 100 infectious disease outbreaks and other health emergencies annually, resulting in unacceptably high morbidity, mortality, disability and socioeconomic losses [4]. Despite the availability of frameworks and strategies, such as the International Health Regulations 2005 (IHR) [5], the African Region’s Integrated Disease Surveillance and Response (IDSR) strategy [6] and the Disaster Risk Management strategy [7], tackling outbreaks and health emergencies continues to be challenging. This is largely due to fragmented implementation of the interventions, limited intersectoral collaboration, inadequate resources, weak health systems, inadequate IHR core capacities and inadequate community engagement in epidemic preparedness and response, resulting in resistance. The foregoing are impediments to attaining Universal Health Coverage (UHC), health security and ultimately the Sustainable Development Goals (SDGs) in many African countries.

To discuss these challenges and propose a way forward for improving health service delivery in the region, the World Health Organization Regional Office for Africa (AFRO), in collaboration with the Government of Cabo Verde, convened the second Africa Health Forum (the Forum) in Praia, Cabo Verde on 26–28 March 2019, under the theme Achieving Universal Health Coverage and Health Security: The Africa We Want to See. The objectives of the Forum, among others, were to discuss innovative strategies to address the persistent public health challenges in the region and to reinforce country ownership and governance for health towards achievement of UHC, health security and the SDGs. The Forum was also an opportunity for Member States^1^ and partners to contribute to reforming the work of AFRO to fulfil the aim of the Africa Health Transformation Programme 2015–2020 [8].

The World Health Organization (WHO) Africa Health Forum was established in 2017 as a platform to strengthen collaboration between WHO and its stakeholders on Africa’s health agenda and to facilitate engagement with all partners to promote partnerships outside of statutory forums, such as the AFRO Regional Committee, which is a governing mechanism involving health ministers of Member States. The Forums are all-inclusive platforms for dialogue towards further development and adoption of joint innovative strategies for improving the health of Africans. The first Forum, under the theme Putting People First: The Road to Universal Health Coverage in Africa, took place in Kigali, Rwanda in June 2017 [9]. This Forum, which brought together African thought leaders, policy-makers and bright young people, adopted the Kigali Call to Action, which reaffirmed the commitment of participants to “putting people first, promoting synergies and coordination and engaging all stakeholders behind the goal of achieving UHC while leaving no one behind”. The second Forum built on the first call to action and recommendations.

In this paper, we present the proceedings and conclusions of the second Forum and propose a number of steps for translating its recommendations and those of the first Forum into concrete actions.

## Methods

The Forum was organized into four main sessions that explored various aspects of UHC, health security and the SDGs in Africa. It was conducted through plenary opening and closing sessions; technical sessions consisting of high-level, moderated panel discussions on specific themes, some of them preceded by a keynote address; and booth exhibitions by Member States, WHO and other organizations to facilitate information exchanges. The speakers included a range of African and international leaders in health policy and international development, drawn from government, the private sector and international organizations [see Additional file 1].

Participants also engaged in daily physical activities and field visits to encourage learning on reforms that are being successfully implemented in Cabo Verde, such as the primary health care network, telemedicine and the intersectoral approach to improve physical activities for the control of non-communicable diseases. A Communiqué highlighting the Forum recommendations was issued at the end of the three-day meeting***.*** Side events on specific topics were organized by Member States, partners and other stakeholders.

More than 750 participants attended the Forum, including leaders and policy-makers, advocates, implementers and partners from sectors with varied affiliations, such as government ministries, donor partners, United Nations agencies, nongovernment organizations, the private sector, academia, youth activist groups and the media. A team of rapporteurs, including some of the authors of this paper, captured and documented the highlights of all sessions and events into a report. We reviewed and extracted the relevant information from that report, notes and observations by the authors during the Forum and synthesized them into these proceedings.

### Proceedings

#### Panel discussions

##### Session 1: taking universal health coverage to the next level in Africa: leaving no one behind

Inherent in the SDGs is the recognition of the central role of health in achieving them [10]. UHC is specified in target 3.8 as the overarching aim for all SDG 3 targets, implying that their attainment should lead to UHC, with the opposite also being true: achieving UHC would lead to the attainment of the other SDG 3 targets. Attaining UHC requires innovative approaches to achieve the outcomes of all health services, for all people, in all situations, for which the current approaches to health care in Africa are not designed. This session explored the overall strategy for addressing UHC in the context of the SDGs and the ways to overcome hindrances to attaining effective UHC results in African countries.

The panellists in this session highlighted the actions that need to be undertaken to make UHC attainable. These include expanding the range of essential health services available in each country, for each age group, building resilient health systems, addressing equity by ensuring that no one is left behind, making available disaggregated data (statistics), information and knowledge for UHC; eliminating financial barriers to accessing health care, promoting multisectoral collaboration and ensuring structured engagement by the private sector. These, they said, should be done without compromising equity and quality.

Several options for the sustainable funding of the foregoing were proffered. These included mobilization of more domestic resources for health through evidence-based justification of the list of services that require funding, the beneficiaries and the return on investments. Also recommended was the more efficient use of available resources and advocacy – with financing institutions like the US President’s Emergency Programme for AIDS Relief, Gavi the Vaccine Alliance, the Global Fund to Fight AIDS, Tuberculosis and Malaria, the Bill and Melinda Gates Foundation, the United States Agency for International Development – on the value of investing in UHC rather than funding vertical, specific disease control and elimination programmes. Other non-financial ways, such as eHealth, to advance the UHC agenda in Africa were also proposed. The role of telemedicine in bringing equitable health services to the nine islands of Cabo Verde was illustrated as an example of how technology can facilitate the attainment of UHC.

Several critical issues emanated from the plenary session: The cost of essential medicines is a barrier to the attainment of the UHC goal, thus regional and national capacities for the local production of essential drugs should be strengthened in Africa. The role of health promotion in the attainment of UHC is critical, and yet, little attention was paid to this in the recent past; countries should thus invest in strengthening the capacity of their citizenry to better understand health issues so that they take their own actions to prevent illness and ensure their well-being. The role of nongovernment organizations, civil society organizations and non-state actors in the attainment of UHC was also emphasized.

The session concluded that UHC is a dynamic and complex process that unravels in different ways in each country; thus, there is a need for an African perspective on UHC that is cognizant of the regional peculiarities, that prioritizes innovative service delivery approaches and that has a long-term perspective. Accelerating the strengthening of national health systems while focusing on the primary health care strategy as the preferred pathway is crucial for achieving UHC, as reiterated in the Astana Declaration on Primary Health Care [11]. Recommendations from the session included the need to facilitate active community participation in health services provision, mobilize additional funding and improve the quality and efficiency of health investments as means to accelerate the attainment of UHC. The proactive generation and use of good-quality data required to monitor progress towards UHC and the abolition of out-of-pocket payments at service points to reduce financial barriers to access services were also stressed.

##### Session 2: multisectoral collaboration to improve health outcomes

The SDGs consist of 17 closely knit goals and 169 targets that are all inclusive, broad and people-centred and based on the principle of leaving no one behind [10]. The achievement of many of these goals and targets has direct and indirect bearing on each other, especially the health goal. Thus, there is considerable need for strong and effective collaboration among all sectors. However, such collaboration is hampered by lack of political commitment, rigid ministerial demarcations that prevent collaboration, inadequate communication between sectors, competition between ministries for funds and relevance, and the culture of vertical programming. This session discussed the various strategies and experiences in effective multisectoral coordination, which can eliminate policy implementation barriers, facilitate scale-up and increase the impact that one sector or partner has on others.

The Health in All Policies (HiAP) approach [12], a strategy that was developed by WHO to support its Member States, was presented as an example of how to use the multisectoral model to improve health outcomes. The approach is a structural partnership in which the interests of all stakeholders are taken into account for mutual benefit. As a result of WHO advocacy, HiAP was adopted by the United Nations General Assembly in 2017 and the African Union in 2019 as instrumental for creating policy coherence across sectors in the pursuit of the SDGs. Several practical examples of the use of HiAP to improve health outcomes at the country level were presented and discussed. Among the examples, Cabo Verde’s Healthy Cities Initiative brought together 22 municipalities and various sectors to collaborate in several areas, such as the promotion of smoke-free cities, consumption of healthy diets, reduction of the harmful use of alcohol, increased physical activity, road safety, improving water and sanitation and waste management. The initiative also included cultural elements and the promotion of happiness through music festivals, health tourism and school health programme. In Zambia, HiAP was used to promote the integration of health into public policies across all sectors; by so doing, the health and health systems implications of decisions made by the various sectors were systematically taken into account to avoid harmful health impacts and ensure synergy across sectors. This perspective has since significantly contributed to prevention and control of cholera and non-communicable diseases in the country.

The session concluded that multisectoral collaboration is critical for meaningful progress towards achieving UHC and health security. Thus, there is need for investment in the promotion of strategies, like HiAP, that would facilitate such collaboration. Countries were encouraged to generate and share evidence to show that effective multisectoral actions lead to positive health outcomes. WHO and its partners were tasked to engage and advocate to Heads of State and Government in championing a systematic and coherent multisectoral agenda for addressing the determinants of health in their countries.

##### Session 3: moving from rhetoric to evidence-based engagement of the private sector for universal health coverage

In view of the ambitious scope of UHC and the SDGs, substantial investments, innovations and new partnerships are required for their attainment. While the public sector has a significant role in leading actions for UHC and SDGs, there is need to engage other partners, including the private sector, to mobilize the necessary capital and harness innovations to support service delivery so as to leave no one behind. While the first Forum and many other international forums have recognized the role of the private sector towards attainment of UHC, actions to facilitate greater private sector involvement in health have not matched the rhetoric. This session therefore explored ways to address the hindrances to attaining effective private sector engagement in the efforts towards UHC and the SDGs in African countries.

The keynote address, which set the tone for the discussion, highlighted that countries that have made significant progress towards UHC and SDGs are those that have relied heavily on public spending. In Africa, however, public resources are limited, with many countries facing shortages of critical cadres of health workers and at least 400 million people having poor access to safe and affordable medicines. To address these gaps, governments need to take advantage of the opportunities that the private sector offers. Such advantages include innovations for health care delivery, networks for extending service coverage and access to medicines and other medical commodities and financial protection. The keynote address was followed by the launch of an AFRO report entitled, *A Heavy Burden: The Indirect Cost of Illness in Africa* [13]. Evidence from the report shows that Africa lags behind the rest of the world in domestic resource mobilization and that spending on health is decreasing in several lower-income countries. The report also showed that while some countries are becoming richer, they are not necessarily spending more on health. The persistent gaps in health funding therefore require a strong case for greater engagement of the private sector. The session reflected on what the private sector could do to support the efforts to achieve UHC.

Three speakers from the private sector showcased how their companies are contributing to the realization of UHC. In Kenya, the health care division of General Electric partnered with the Government to renovate and equip 98 health facilities with low-cost diagnostic equipment [14]. The partnership also included transferring know-how for the maintenance and use of the equipment. This Public-Private Partnership (PPP) resulted in 700 health workers being trained annually and has supported the construction of an oncology centre in Kenya. The partnership also resulted in 50% improved access to care and reduced patient costs by half. Also in Kenya, PharmaAccess Foundation partnered with banks and governments to provide simple and affordable options for improving access to health care using mobile wallets [15]. The Foundation uses the M-Pesa platform developed by Safaricom, a private telecommunications company, to pool different types of funding and to provide capital to small and medium-sized health facilities to deliver services. This partnership now involves some 2000 local clinics that reach about two million clients every month. In South Africa, the BIOVAC Institute is engaged in a PPP with the Government to support the development of safe and affordable vaccines [16]. The partnership is funded through loans from banks and partners, such as the Programme for Appropriate Technology for Health. This innovative approach has resulted in technology transfer from Sanofi and Merck in the labelling and packaging of essential medicines.

The panellists acknowledged that African governments cannot solve all their health problems alone, thus they should provide a conducive environment and legal framework for the effective engagement of the private sector. The panel discussion also reflected on how private sector actors could be organized to engage better with governments towards UHC. The need for each stakeholder to better understand their respective roles and how the other works as well as the role of the public sector in enabling, coordinating and boosting partnerships with the private sector were highlighted. Participants called for a continental approach to regulatory mechanisms, including marketing and pricing, registration and quality for essential medicines. They also called for a change of mindset at the country level, whereby governments and partners support indigenous and local companies to manufacture medicines and vaccines. They requested governments to implement preferential purchasing policies that target local manufacturing in order to stimulate growth in the domestic pharmaceutical sector.

The session concluded with recognition that enhanced engagement with the private sector requires enabling legal, policy and regulatory environments and building trust between the private and public sectors through continuous dialogue. African governments were tasked to identify suitable areas for engaging and contracting the private sector to expand service coverage and to develop adequate frameworks to ensure mutual transparency and accountability. The private sector was called upon to leverage their resources to support innovative approaches towards attainment of UHC and health security in Africa, while WHO and other United Nations partners were tasked to provide the required technical support for the design of PPPs and to generate evidence on best practices in public-private engagement.

##### Session 4: health security in Africa: from preparedness to response – collaboration for improved coordination, preparedness and global health security

The need for strong partnerships and collaboration to ensure health security in the WHO African Region cannot be overemphasized. The numerous health security initiatives, projects, meetings, trainings and workshops in Africa require better coordination and a common regional platform that allows them to connect, share and collaborate for better coordination to improve health security. This session brought together national, regional and international stakeholders to share their collective success towards developing the IHR core capacities and to discuss how multisectoral partnerships for health security can be improved in Africa. The session was divided into four sub-sessions that looked at various aspects of health security in the WHO African Region.

##### Sub-session 1: health security in Africa – from preparedness to response

This session discussed the ongoing Ebola Virus Disease (EVD) outbreak in the Democratic Republic of the Congo and highlighted efforts to overcome the challenges, including the insecurity situation in the affected eastern region. In the keynote address, the impact of disease outbreaks on Africa was highlighted; this includes loss of life, livelihoods and financial resources – in billions of US dollars. These losses could be prevented by early detection and response to such outbreaks. Early detection and effective outbreak response require that all African countries develop and implement a national action plan for health security.

The EVD outbreak in eastern DRC is the tenth in the country and the second-largest outbreak recorded globally. At the time of the Forum, the outbreak had affected 20 health districts, from which 1019 EVD cases and 637 deaths had been reported [17]. The challenges faced include the high population density, insecurity, community resistance, lack of trust and weak community engagement. Ongoing response efforts include ring vaccination, with more than 90,000 persons vaccinated (as of the time of the Forum), contact tracing, surveillance, case management and community mobilization.

The session concluded that the response to the outbreak should include not only public health but also social and gender-sensitive interventions and that the lessons learned from the 2014–2016 EVD outbreak in West Africa should be applied to inform prevention and control of the current outbreak. These lessons include using social anthropologists and sociologists to better understand the perception of risk and to better design specific community engagement strategies. Strategies to address the problem of insecurity, such as neutrality of the response teams and intense dialogue with the communities, were also proposed.

##### Sub-session 2: opportunities and challenges for health security in Africa

Although significant investment has been made in implementing the IHR, all countries in the WHO African Region regrettably have varying capacities to quickly detect and rapidly and effectively respond to public health events. This is despite the fact that 85% of African countries have conducted Joint External Evaluations to assess their IHR core capacities and 50% have a multisectoral national action plan for health security [18]. The Joint External Evaluations showed that, except for some progress made in immunization, surveillance and laboratory capacity at national level, all the remaining technical areas of IHR core capacities are still very weak. This session therefore explored the opportunities and challenges in achieving health security in Africa as a means to achieving UHC.

The keynote address reiterated the central role of communities in the response to crises, emphasizing that “outbreaks start in communities and end with communities”. The solidarity that is built by communities to support themselves during crises should be harnessed to mount community-centred responses, which are built on social norms and social cohesion. But harnessing community potential must be based on trust and long-term partnership, which ensures community participation in public health interventions in the pre-, intra- and post-crises periods. The role of governments in strengthening the IHR core capacities to protect their populations and prevent public health emergencies of international concern was also emphasized.

Other opportunities for enhancing health security in Africa were presented and deliberated during the panel discussion. The role of information and communication technology in strengthening surveillance of diseases of epidemic potential was highlighted. For example, a simple cell phone is a good tool for surveillance that can be used by health facilities or communities because it helps receive and send information in real time and provides quick information for decision-making. Big data technology can also be used to manage big data sets, which quickens the analysis process, thus ensuring that data are available for decision-making in a timely manner [19]. Drones were used to conduct rapid health assessments in communities affected by Cyclones and used to prioritize areas that required targeted humanitarian assistance [20, 21]. Reaping the potential benefits associated with these technologies requires mobilization of the private sector, which has the technological know-how.

The critical role of women, culture and religion in the effective response to health issues was also highlighted. The pre-emptive use of anthropology to better understand community norms, cultural and religious beliefs was cited as a good approach to develop and implement community-specific outbreak strategies and interventions.

The session concluded that although significant investments have been made in implementing the IHR monitoring and evaluation framework, the African Region is still far from attaining the critical IHR core capacities as well as resilient health systems, which are required for attaining health security. Member States therefore need to allocate adequate domestic funds and continually mobilize additional resources from international and national partners for addressing the IHR capacity gaps articulated in their national action plan for health security. Other messages from the session included the need to invest in empowering and building the capacity of communities to identify their priorities and risks and to effectively respond to them. Proactive engagement of the private sector to mobilize resources and to support the use of technology for enhancing health security in Africa was also recommended.

##### Sub-session 3: health security as an entry point to universal health coverage

The session explored the contributions of health security to UHC. In the keynote address, entitled “The Contribution of Health Security to Universal Health Coverage”, the work of the Lancet Commission on Synergies [22] was introduced. The Commission was established in 2018 with the aim of finding synergies between UHC, health security and health promotion and to provide evidence and information to policy-makers that will align their efforts to achieve these goals. The Commission is of the view that the three constitute the three top-most priorities for global health, a view that is corroborated by the WHO Thirteenth Global Programme of work and its triple-billion targets for promoting health, keeping the world safe and serving the vulnerable [23].

The links between the three strategic priorities and how they can work synergistically were emphasized. Stronger health systems are required for UHC and health security, while efforts towards strengthening UHC could contribute to improving health security, and vice versa. Furthermore, population-based prevention interventions can also contribute to both health security and UHC. To promote synergies and avoid fragmentation, the Lancet Commission focuses on identifying interventions that will contribute to each of these priorities and where making progress in one area amplifies progress in the others; this would help stakeholders to better align their efforts and resources. For example, large outbreaks can overwhelm health systems and make the provision of normal health services impossible, at least temporarily, as was the case during the 2014–2016 West Africa EVD epidemic, in which more people died from lack of access to regular health services than from the Ebola virus [24]. In addition, the financial impact of the Ebola response reduced the capacity of the affected countries to invest in their health system in the long term [25]. This shows that investment in health systems is needed to protect the population from the devastating impact of outbreaks [26, 27]. To achieve UHC, there is urgent need to invest in a range of specialized and basic health services that includes public health interventions, periodic outreach services, health information and capacity for analysis, all of which are also critical for health security. UHC and health security therefore need to be brought closer together to synergize each other.

The panel discussion reflected on the messages from the keynote address. The need for innovations that can ensure sustainable financing of health security and UHC interventions was articulated, using the example of an air ambulance service in Nigeria as a good solution. The air ambulance service worked with local Nigerian airlines to design a small cabin that could be used to provide intensive care in the economy class of a commercial flight, bringing down the cost of a medical evacuation flight from US$25,000 to about US$500.

The session concluded that strong health systems are the cornerstone for UHC and health security and called for effective intersectoral collaboration to achieve the three global health priorities of UHC, health security and health promotion. The SDGs should be used as an opportunity for African countries to target the global health priorities in a synergistic manner. Cost-effective innovations and local solutions for UHC and health security that can exponentially decrease the costs of health care are required.

##### Sub-session 4: sustainable financing for health security in Africa

This session looked at successful approaches and new opportunities for financing health security at the subnational, national, subregional, regional and global levels. The keynote address on “Domestic Financing for Sustainable Health Security in Africa” highlighted the paradox for African islands: Due to their size, they quickly achieve good health care outcomes but pay a premium price per capita when it comes to investing in health systems because they lack economies of scale. In addition, they are the most vulnerable to external environmental or economic shocks because, like other countries in Africa, they do not have a resilient public health system to respond quickly and effectively to crises. The Seychelles example, however, demonstrates that, costly as it may be, UHC is critical to ensuring health security. By delivering on primary health care, the country has built more resilience to external shocks. Also highlighted in the session is the need to continuously identify new sources of financing for health, including national insurance systems and taxation on alcohol, cigarettes and sugar-sweetened beverages, which can have a deterrent effect on consumption while raising revenue.

Various modalities for sustainable financing for health security in Africa were presented and discussed during the panel discussion. Since the late 1970s, the African Development Bank, for example, has been supporting African countries in the areas of health security and UHC, using both sovereign and non-sovereign lending windows. In the past few years, the Bank has supported countries to respond to outbreaks and epidemics, including the 2014–2016 EVD epidemic in West Africa, to which the Bank committed more than US$200 million. However, a critical lesson learned is that some countries lack the capacity to absorb the resources provided for health issues within the agreed time frame. To address this, the Bank has incorporated knowledge transfer, capacity building and sharing of lessons into its support to Member States.

In Zimbabwe, Econet Wireless, a private mobile communications company, supported the Government to respond to the 2018–2019 cholera outbreak through its philanthropic arm, the Higherlife Foundation [28]. The Foundation is supporting the Ministry of Health in surveillance and establishment of a public health emergency operation centre to coordinate emergency preparedness and response. The lessons from this experience are that the private sector has some comparative advantages, such as skills, functional systems and finances, which governments could leverage for increased financing of health security.

Other innovative financing schemes, such as health insurance and taxation to improve health security, also exist on the continent. For example, a South African-based organization, Africa Risk Capacity, works with WHO Member States to model their risks and then provides insurance that governments can buy to respond to epidemics. Using this model, four countries have been supported in responding to droughts, to the tune of US$37 million [29].

The session concluded that all relevant stakeholders, including the private sector, should come together to build strong partnerships to address the current financing gap for health security on the continent. To do this, ministries of health should make a strong case to ministers of finance for increased investment in health security and UHC in a predictable and efficient manner.

#### Side events

Six side events with themes that complemented the overall theme and discussion at the Forum took place.

##### Side event 1: investing in young people as a key to achieving universal health coverage in Africa

Youths should be engaged and empowered in order to reap the benefits of the demographic dividend, which could contribute to the attainment of UHC and the SDGs. This side event focused on the progress made in empowering African youths and how to strengthen institutional partnerships and participatory approaches that enable young people assume leadership in issues affecting their health in Africa. The event concluded that countries should establish adolescent-friendly health services to achieve UHC and better life expectancy for young people and to ensure that adolescents and youths are considered as key partners and actors in developing, implementing, monitoring and evaluating national health policies and strategies. A call to create an environment conducive for adolescents and youths to exercise their rights and achieve their full potential through access to good health services, education, training, technologies and employment was also made.

##### Side event 2: best practices for universal health coverage from Cabo Verde

Cabo Verde continuously encounters structural challenges in the organization of health services due to the scattered nature of its islands. Nevertheless, it has been able to improve health care, reduce maternal and infant mortality, increase immunization coverage and increase life expectancy through the decentralization of health care provision to the municipalities and communities. This side event showcased the experience and best practices of Cabo Verde in the achievement of UHC. Implementation of a number of national programmes to address priority health issues, such as non-communicable diseases (Programme Mexi-mexe and the Presidential Initiative on More Life, Less Alcohol), prevention of HIV transmission from mother to child and reduction of childhood mortality have resulted in improved health outcomes in the country. The gains made from these programmes contributed substantially to the development and social cohesion of the country and to its graduation to a middle-income country. The event concluded that fundamental practices, such as identification of health priorities, decentralization of health services, establishment of a rigorous system for measuring the impact of health interventions, and political commitment, are critical for attaining UHC in Africa.

##### Side event 3: celebrating 20 years of integrated diseases surveillance and response strategy in Africa

The IDSR strategy was developed in 1998 by AFRO and its partners to support implementation of public health surveillance and epidemic response systems at all levels of the health system in Africa. Over the years, the strategy has been reviewed to incorporate emerging topics, such as the IHR, new diseases, conditions and events, non-communicable diseases and community-based disease surveillance. The third edition of the IDSR strategy, which was developed in 2019, is being implemented in the WHO African Region. This side event publicized the achievements and challenges of IDSR implementation over the past 20 years in Africa. Forty-four of the 47 African Member States of WHO are implementing the strategy, while the remaining three have signified their intention to adopt the latest version [30]. A recent assessment of the strategy found significant improvement in the timeliness of outbreak detection and a shortened response time as a result of its implementation. With the strategy in place, outbreaks that would have taken several days to control are now being detected and managed within a shorter period of time. The event concluded by encouraging all African countries to adapt and implement the third edition of IDSR and to explore innovative and sustainable ways to fund its implementation, with WHO and relevant partners called upon to continue advocating for increased investment in IDSR.

##### Side event 4: cholera prevention and control

Cholera remains a major public health problem in Africa. In 2017, an estimated 179,000 cases, including more than 3220 deaths, representing a case fatality ratio of 1.8%, were reported from 14 countries of the WHO African Region [31]. Globally, about 1.3 million cholera cases and 143,000 deaths from cholera are reported annually [32]. This side event focused on the progress made in the implementation of the Regional Framework for the Implementation of the WHO Renewed Strategy for Cholera Prevention and Control, 2018–2030 [33], with a view to renew the strong regional partnership towards the elimination of cholera in the African Region. The session concluded that outbreaks of cholera are a failure of the health system and require a three-prong approach for prevention and control – early detection and quick response; the adoption of a multisectoral approach for prevention, with more emphasis on water and sanitation; and a strong coordination mechanism with strong partnerships. African countries were tasked to ensure implementation of the regional framework and to facilitate collaboration between various partners to support the implementation of national action plans for cholera.

##### Side event 5: global strategic preparedness network for Africa

In 2005, Member States, partners and donors asked WHO to develop the Global Strategic Preparedness Network (GSPN) as a platform for coherent and coordinated approach for providing technical support to countries for emergency preparedness. This network involves support to countries to coordinate the deployment of technical expertise, to strengthen preparedness networks by sharing expertise and good practices and to implement their national action plan for health security. This side event discussed how AFRO can work as the convener of health emergency preparedness in the region using the GSPN model. The GSPN was adjudged a timely initiative and likened to the Global Outbreak Alert and Response Network (GOARN), which is a successful collaboration of institutions and networks that pools human and technical resources for rapid identification, confirmation and response during outbreaks of international importance. While GOARN is designed for response, GSPN should focus on coordinating the technical expertise needed for preparedness. Participants at the event called for further consultations with African countries on the network’s development so as to foster country ownership. The event concluded by recommending that WHO should continue to consult and solicit feedback from African countries and other stakeholders on the GSPN and to increase advocacy and awareness about the initiative.

##### Side event 6: geographic information system technology for polio surveillance

One of the key innovations that can drive attainment of UHC is the application of technology to health. This side event showcased the versatility and real-time capabilities of the AFRO-developed Geographic Information System (GIS) technology, and its adaptability for other health interventions beyond polio, including contributing to UHC. The technology was designed by AFRO in 2017, with funding from the Bill and Melinda Gates Foundation, to enhance polio surveillance and immunization activities in the field. It is also used to support other interventions such as immunization, response to outbreaks of cholera, meningitis, measles and Lassa fever, as well as for conducting surveys to evaluate measles campaigns, immunization coverage and mortality in inaccessible and hard-to-reach areas. The technology is relatively cheap, requires minimal investment (an android-driven cell phone) and is sustainable beyond the polio eradication initiative. The technology has been adopted and implemented in 43 of the 47 African Member States of WHO. It has now been expanded to cover more than 5000 community informants in hard-to-reach and inaccessible areas of Africa. The session concluded by recommending that the GIS technology should be made interoperable with other electronic tools, such as the electronic IDSR system, the District Health Information System 2 and the Outbreak Response Management System. African countries were called upon to put in place mechanisms to ensure the sustainability of this technology, with AFRO urged to continue providing the required technical support to countries to implement other GIS-based innovative interventions for UHC.

#### The innovation challenge

Innovations are key drivers of new achievements in Africa, particularly for UHC, health security and the SDGs, which was a central and cross-cutting theme for the Forum. An innovations exhibition centre was on display throughout the Forum to showcase the top 30 health innovations selected from the inaugural AFRO Innovation Challenge, which was launched in October 2018. The Challenge sought to source and profile health innovations that could be sustainably scaled up to improve health outcomes and quality of life and to offer solutions to unmet health needs in Africa. Discussions during all the Forum sessions underscored the need to ensure that support is provided for scaling up the innovations that were submitted to the Challenge. Members States were urged to develop and implement innovation-friendly policies and strategies, provide fiscal and non-fiscal incentives to support the development of health innovations and to institutionalize the use of GIS technological innovations to monitor and accelerate progress towards UHC, including preparing for and responding to health emergencies. WHO, other partners and the private sector were called upon to support the scale-up of health innovations in the region and to compile and share good practices in health innovations across the continent.

#### Communique

The second Forum concluded with the issuance of a Communique that summarized the recommendations, to which the participants committed themselves to following through on. The Communique also aims to fast-track the implementation of the Kigali Call to Action, which was agreed upon at the end of the first Forum [see Additional file 2].

## Conclusions

The Forum has established itself as a credible platform for analysing, discussing and proposing home-grown solutions to Africa’s public health challenges. It has also become a strategic and all-inclusive platform for discussing modalities to domesticate global development and humanitarian initiatives to the regional context. A key strength of the Forum is its multidisciplinary approach, which brings together and gives equal voice to practitioners drawn from the public and private sectors and civil society. This is appropriate for the African context and in line with new paradigm shifts bringing together experts from different fields of health, economics, sociology, anthropology and other fields for holistic discussion on solving the public health problems of Africa.

Reaping the full benefits of the Forum requires moving from rhetoric to deed by establishing sustainable mechanisms for translating the recommendations of the Forum into action. Such mechanisms should include strategies for collective operational planning, implementation, supervision, monitoring and evaluation of the recommendations of all the Forums.

Many critical insights for supporting the attainment of UHC, health security and ultimately the SDGs in Africa emerged from the second Forum. First, the role of community engagement and participation in the attainment of UHC, health security and the SDGs cannot be overemphasized. Community participation in the planning, implementation and monitoring of health programmes is critical for breaking down silos, preventing community resistance and ensuring uptake of health services, all of which would contribute to UHC, health security and the SDGs.

Second, the Forum recognized that the public sector of Africa cannot singularly attain the three interrelated goals of UHC, health security and the SDGs, thus other partners, such as the private sector, need to be engaged to harness their comparative advantages in innovation, technology and financing. While this has been recognized in several forums, there is need to move from words to action by establishing mechanisms to encourage increased private sector involvement in health in the region. Beyond the discussions and recommendations of the second Forum, the civil society (although still weak in many Africa countries) could fill critical roles in ensuring transparency and accountability in community and private sector engagement.

Third, technological innovations will be a key driver in attaining UHC, health security and the SDGs. Hence, there is need to harness the comparative advantages that they offer. However, such technological innovations should be sustainable and fit for the African purpose. Fourth, attainment of UHC, health security and the SDGs are intertwined. Attainment of one paves the way for the others; hence, there is need for integrated and synergistic public health approaches in planning for and implementing interventions aimed at achieving the three goals. Fifth, despite the critical role of women and gender in the attainment of the three interrelated goals, there was no dedicated session on gender during the second Forum; hence, there is need for greater attention to this topic in the next Forum.

## Recommendations

To ensure that the recommendations of both the first and second Forums are translated into concrete actions in a sustainable manner, we call on African Ministers of Health to ensure their integration into national health policies, strategic and other health sector documents and to provide the necessary leadership and enabling environment required for their implementation. AFRO and its country offices should ensure that, where appropriate, the recommendations are incorporated into their country cooperation strategies and biennial programme budget as part of their ongoing technical assistance to the Member States. We also call upon partners and other stakeholders to mainstream these into their ongoing support to African Member States. Finally, AFRO should support Member States to establish a system to track and report on the progress made and challenges encountered in the implementation of the recommendations so as to institute timely remedial actions.

## Supplementary information

**Additional file 1.** Annotated programme of the second WHO Africa Health Forum.

**Additional file 2.** Communique of the second WHO Africa Health Forum.

## Data Availability

The materials and information used in the preparation of this manuscript were extracted from the final report of the second Forum, which is readily available at the Forum website.

## Abbreviations

AFRO: World Health Organization Regional Office for Africa
EVD: Ebola Virus Disease
GIS: Geographic Information System
GOARN: Global Outbreak Alert and Response Network
GSPN: Global Strategic Preparedness Network
HiAP: Health in All Policies
IDSR: Integrated Disease Surveillance and Response
IHR: International Health Regulations 2005
PPP: Public-Private Partnership
SDGs: Sustainable Development Goals
The Forum: Second WHO Africa Health Forum
UHC: Universal Health Coverage
WHO: World Health Organization

## Footnotes

1 In the context of this article, WHO Member States, African Region and African countries refers to the 47 Members States of AFRO: Algeria, Angola, Benin, Botswana, Burkina Faso, Burundi, Cabo Verde, Cameroon, Central Africa Republic, Chad, Comoros, Cote d’Ivoire, Democratic Republic of the Congo, Equatorial Guinea, Eritrea, Ethiopia, Gabon, the Gambia, Ghana, Guinea, Guinea Bissau, Kenya, Lesotho, Liberia, Madagascar, Malawi, Mali, Mauritania, Mauritius, Mozambique, Namibia, Niger, Nigeria, Republic of Congo, Republic of South Sudan, Rwanda, Sao Tome and Principe, Senegal, Seychelles, Sierra Leone, South Africa, Swaziland, Togo, Uganda, United Republic of Tanzania, Zambia and Zimbabwe.
