# Correction to: Community participation and private sector engagement are fundamental to achieving universal health coverage and health security in Africa: reflections from the second Africa health forum

**DOI:** 10.1186/s12919-019-0182-9

**Published:** 2019-12-02

**Authors:** Olushayo Olu, Pamela Drameh-Avognon, Emil Asamoah-Odei, Francis Kasolo, Tomas Valdez, Grace Kabaniha, Humphrey Karamagi, Suvajee Good, Helena O’Malley, Zabulon Yoti, Nirina Razakazoa, Etienne Minkoulou, Jean-Marie Dangou, Symplice Mbola Mbassi, Mariano Salazar Castellon, Joseph Cabore, Matshidiso Moeti

**Affiliations:** 1 WHO Country Office, Juba, Republic of South Sudan; 2 WHO Regional Office for Africa, Brazzaville, Republic of the Congo; 3 Consultant, Regional Director’s Office, WHO Regional Office for Africa, Brazzaville, Republic of the Congo; 4 WHO Country Office, Praia, Cabo Verde

**Correction to: BMC Proc (2019) 13:7**

**https://doi.org/10.1186/s12919-019-0170-0**

From Second WHO Africa Health Forum

Praia, Cabo Verde. 26-28 March 2019

Following publication of the original article [1], the authors reported the following author name error is the article:

Incorrect: Thomas Valdez

Correct: Tomas Valdez
