# Supplementary material for: Community participation and private sector engagement are fundamental to achieving universal health coverage and health security in Africa: reflections from the second Africa health forum
Source: BMC Proc. 2019 Nov 12;13(Suppl 9):7. doi: 10.1186/s12919-019-0170-0 (PMC6849158; doi:10.1186/s12919-019-0170-0)
Supplement: Supplementary file 1 — Additional file 1. Annotated programme of the second WHO Africa Health Forum. [file 12919_2019_170_MOESM1_ESM.pdf]

THE 2ND

WHO AFRICA HEALTH FORUM

Achieving Universal Health Coverage and Health Security: **The Africa We Want to See**

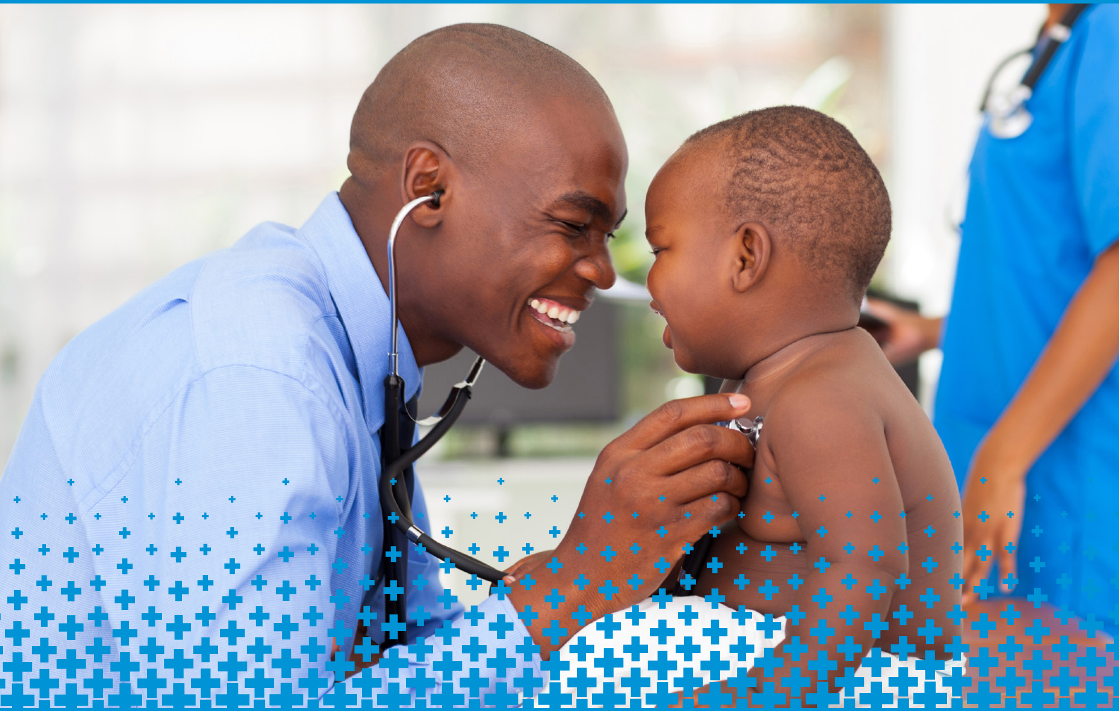

MINISTÉRIO DA SAÚDE E DA SEGURANÇA SOCIAL

GOVERNO DE CABO VERDE
A TRABALHAR PARA TODOS.

World Health Organization
REGIONAL OFFICE FOR Africa

Welcome to the second WHO Africa Health Forum!
I am delighted you have joined us – it is our strategic partnerships, effective engagement and coordinated joint actions that will ultimately result in the better management and mitigation of the urgent and ever-changing health needs of African people.

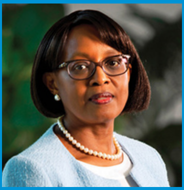

We all know that Africa's health challenges have become increasingly complex. With the continent facing a growing number of disease outbreaks and the dual challenges of communicable and non-communicable diseases, we cannot continue with business as usual.

The theme for this Forum is "Achieving Universal Health Coverage and Health Security: The Africa We Want to See." It underscores how good health for all, including ensuring health security and universal health coverage, is central to the continent's development and why the Government of Cabo Verde and the World Health Organization Regional Office for Africa are jointly hosting the second WHO Africa Health Forum.

During this meeting in Praia, we will also be reaffirming the commitment we made in the first WHO Africa Health Forum in Kigali, Rwanda, two years ago: putting people first, promoting synergies and coherence and engaging all stakeholders behind the goal of achieving universal health coverage while leaving no one behind.

This second Forum will introduce innovations and kick-start new partnerships that can reshape how we practise health development. It will also emphasize the need to optimize multisectoral partnerships for Universal Health Coverage and Health Security.

I trust that you will find this Forum engaging and that it will carry on the fruitful partnering we started with the first Forum. Enjoy your stay in Cabo Verde, and we look forward to your continued and needed engagement and collaboration.

Yours sincerely,

Dr Matshidiso Moeti
WHO Regional Director for Africa

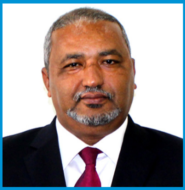

Cabo Verde is honoured and proud to host the second Africa Health Forum, in partnership with the World Health Organization and under the theme “Achieving Universal Health Coverage and Health Security: The Africa We Want to See”.
In hosting this Forum, Cabo Verde reiterates its commitment to contribute to a healthier Africa, in a partnership with all countries in our region and our continent that is focused on people.
Welcome to Cabo Verde, full of morabeza! United and together for a healthier Africa!

Yours sincerely,

H.E. Dr Arlindo Nascimento do Rosário
Minister of Health and Social Security, Cabo Verde

The Government of Cabo Verde and the World Health Organization Regional Office for Africa are jointly hosting the second WHO Africa Health Forum on the theme: “Achieving Universal Health Coverage and Health Security: The Africa We Want to See”.

The second WHO Africa Health Forum is taking place in Praia, Cabo Verde, 26–28 March and is 2019’s seminal health-focused event on the continent. Together, participants are looking at workable solutions for more effective health security governance, for strengthening health systems and for ultimately delivering universal health coverage. The Forum is expected to help kick-start new partnerships for improving the health of people across the region.

The Forum brings together African leaders, ministers of health and finance, development partners, UN agencies, academics, civil society, philanthropic foundations, the private sector, youth activists and new regional and global health players to exchange experiences and optimize multisectoral partnerships. The focus on educating and engaging the youth is of particular importance.

PRE-EVENT
25 MARCH 2019

08:30–12:30
Visit to the North Santiago Island Health Region (departure: hotels)

Technical office

Santa-Rita Vieira Regional Hospital

Visit to the Health Department of Santa Cruz (departure: hotels)

14:30–16:00
Visit to the Agostinho Neto Central Hospital (departure: hotels)

Telemedicine services

Neonatology services

17:00–18:00
Human solidarity chain for universal health coverage flash mob at the Quebra Canela beach area (departure: hotels)

DAY ONE
26 MARCH 2019

06:30–07:30

Morning walk the talk – Open-air jogging & aerobics at the Quebra Canela beach area (departure: hotels)

08:00

Registration – National Assembly foyer

09:00–10:30

Opening ceremony

Welcome and introductory remarks by master of ceremonies, **Mr Alveno Figueiredo,** Media Advisor, National Assembly of Cabo Verde

Opening address by **H.E. Dr Arlindo Nascimento do Rosário**, Minister of Health and Social Security, Cabo Verde

Welcome address by **Dr Matshidiso Moeti**, WHO Regional Director for Africa

Keynote and welcome address by **H.E. Jorge Carlos Almeida Fonseca**, President of Cabo Verde

10:30–11:00

Launch of the exhibition to showcase health innovations in Africa

Ribbon-cutting ceremony – Launch of the exhibition of health innovation finalists by President of Cabo Verde, followed by a visit to the booths – to be announced by master of ceremonies **Mr Alveno Figueiredo**, Media Advisor, National Assembly of Cabo Verde

National Assembly upper level

African innovations to solve African health challenges

From more than 2 400 applications, the first-ever WHO Africa Innovation Challenge introduces 30 African solutions to the continent's unmet health needs. Calling for scalable health care solutions, the Challenge received a wealth of ideas in three categories: Product Innovation, Service Innovation and Social Innovation. The 30 solutions on display here at the forum were selected by a team of independent expert evaluators. With this Innovation Challenge, says **Dr Matshidiso Moeti**, WHO Regional Director for Africa, WHO is championing the promotion of home-grown solutions to the health challenges in African countries. "We hope this challenge will spark the entrepreneurial spirit of innovators and lead to credible health innovations across the continent."

MODERATOR

Ms Denise Epote, Regional Director for Africa, TV5Monde

Group photo and human solidarity chain

National Assembly upper level

Networking National Assembly lower level

11:00–11:30

Press conference: Sala Jornadas 2

11:30–13:30

Session 1: Taking Universal Health Coverage to the Next Step in Africa: Leave No One Behind – Salão Nobre

Universal health coverage represents an aspiration that countries in the WHO African Region have all committed to achieve. This is a significant shift from the traditional approach to health services, in which focus was on priority populations receiving priority services. Universal health coverage requires innovative approaches to attain the goal of all services, for all people, in all situations – for which the current approaches to health care are not designed.

Guided by the theme, Achieving Universal Health Coverage and Health Security: The Africa We Want to See, the second WHO Africa Health Forum will bring together hundreds of influencers from the region and beyond. Together with the more than 35 panellists – leaders in their fields – the Forum participants will challenge the status quo and explore new directions that should guide countries as they chart their path towards universal health coverage.

PANELLISTS

Dr Prosper Tumusiime, Director a.i., Health Systems Strengthening Cluster

Ms Sofia Moreira de Sousa, European Union Ambassador and Head of the European Union Delegation to Cabo Verde

Ms Loyce Sousa, President & Executive Director, Global Health Council

Dr Luis Gomes Sambo, Regional Director Emeritus, WHO African Region

Dr Leonardo Simão, High Representative Africa, European and Developing Countries Clinical Trials Partnership

MODERATOR

Mr Henry Bonsu, Journalist, International broadcaster

13:30–15:30

Lunch and networking: Restaurant and tent

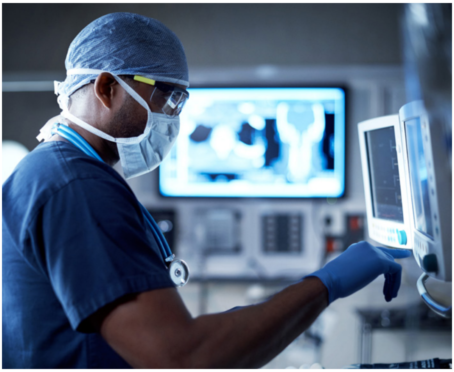

14:15–15:15

Youth side event: Investing in Young People as a Key to Achieving Universal Health Coverage in the African Region – Sala Jornadas 1 and Salão Nobre

The inaugural WHO Africa Health Forum (2017) launched the WHO Adolescent Health Flagship Programme to advocate for greater investment in young people – aged 10–24 years – to achieve universal health coverage in the WHO African Region. In a side event on the first of the three-day gathering, the second Forum will look at the progress made and hear from several youth activists on what more could be done to tackle issues that are imperative to the health of young people and their development and end the persistent challenges in young people-specific public health. Young people will give their perspective on how to hold policy-makers and decision-makers accountable and expand leadership, ownership and involvement of young people in governance and how to achieve young people-responsive health and social systems that move communities towards universal health coverage. "Youth booths" will be available all three days to encourage dialogue with the invited youth activists on health security and new health threats in Africa and young people's role in preparedness, response and equitable access to health services, tackling the social and economic determinants of health and health literacy.

PANELLISTS

H.E. Dr Fernando Elisio Freire, Minister of State, Parliamentary Affairs, President of the Council of Ministers and Minister of Sports, Cabo Verde

Hon. Toussaint Manga, Youth parliamentarian

Ms Natasha Chibesa Mwansa, Youth activist

Mr Mamadou Kante, UNFPA WCA Deputy Regional Director

Hon. Toussaint Manga, Young Parliamentarian

Mr. Awal Issa Rachid, President, Youth Forum, Niamey

MODERATORS

Ms Harriet Yayra Adzofu, Youth Without Borders, and **Mr Ruben Filipe Semedo Ramos**, Youth activist

Final remarks by **Dr Matshidiso Moeti**, WHO Regional Director for Africa

15:30–17:30

Session 2: Multisector Collaboration to Improve Health Outcomes – Salão Nobre

The 2030 Agenda for Sustainable Development, adopted by the United Nations General Assembly in 2015, focuses on ensuring the attainment of development in a manner that is equitable and sustainable. Health is both a determinant and an outcome of sustainable development interventions. It is inextricably linked to other sustainable development outcomes, including better education, higher productivity and, consequently, higher wages in later life. Africa's sustainable development largely depends on goods and services derived from its environment and natural resource base. Agriculture, tourism, industry, mining and many forms of local, national, regional and international trade thrive on the goods and services provided by natural resources. Sustainable development programmes that improve or preserve the quality of water, air and other environmental resources and services will lower national health budgets and ensure a

healthy labour force.

The panellists will bring their insights on how the health sectors: should best interrelate with other development sectors and will analyse multisector collaboration to improve health-related actions since the first WHO Africa Health Forum. They will pinpoint the strengths and weaknesses of the collaboration and discuss how the partnerships can eliminate policy implementation barriers, facilitate scaling up of services and increase impacts. They will also call for a multisectoral coordination platform, led by Heads of State or Prime Ministers, to further the engagement with stakeholders in multisector collaboration to achieve the Sustainable Development Goals.

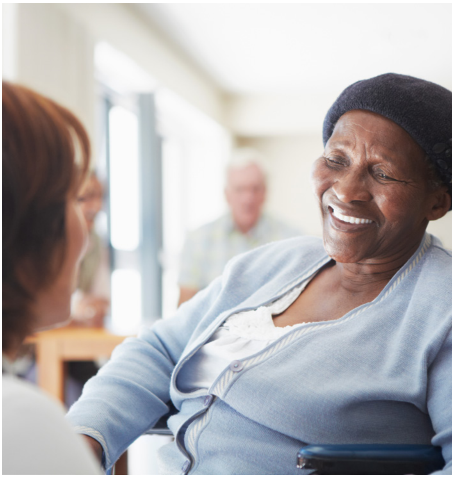

PANELLISTS

Dr Matshidiso Moeti, WHO Regional Director for Africa

Ms Helena Rebelo Rodrigues, Head of Support Unit for the Implementation of the Healthy Cities Initiative, Cabo Verde

Mr Felix Dounia Millimono, Leader, African Youth and Adolescent Network

Mr Abdouraman Bary, Programme Officer, United Nations Environment Programme Regional Office for Africa

MODERATOR

Dr Joannie Bewa, Public health and public policy specialist

18:00

Welcome reception: National Assembly balcony area upper floor

DAY TWO
27 MARCH 2019

06:30–07:30

Morning walk the talk: Open air jogging & aerobics at the Quebra Canela beach area (departure: hotels)

09:00–11:00

Session 3: Moving Beyond Rhetoric to Evidence-based Action for Private Sector Engagement for UHC - Salão Nobre
Presentations to set the scene from Kenya, M-Tiba, South Africa, Senegal and Harvard Medical School

The 2030 Agenda for Sustainable Development calls for an ambitious investment of US$ 2.5 trillion for achieving the Sustainable Development Goals. Such an ambition will take all actors pitching in to generate the capital, undertake innovation for action and enhance service delivery so that no one is left behind any longer in Africa. Arriving at universal health coverage requires joining hands with the private sector, which can only happen within a conducive legislative and regulatory environment and the right type of incentives.

Leaders in industry and philanthropy in Africa and beyond will explore the ideal model for decision-making that can guide Member States on how and when to engage the private sector. They will also explain how the private sector can contribute more strategically to the health value chain and what the private sector needs to increase its engagement with health care.

PANELLISTS

H.E. Dr Olavo Avelino Garcia Correia, Deputy Prime Minister of Cabo Verde and Minister of Finance

Dr Amit Thakker, Chairman, African Health Care Federation and Africa Health Business

Ms Thokozile Ruzvidzo, Director, Social Affairs, United Nations Economic Commission for Africa

Mr Alim Hayatou, Secretary of State, Ministry of Health, Cameroon

Mr Greg Perry, Assistant General Director, International Federation of Pharmaceutical Manufacturers & Associations

MODERATOR

Mr Henry Bonsu, Journalist, International broadcaster

Opening Remarks by Dr Matshidiso Moeti, WHO Regional Director for Africa

Presentations to set the scene from:
Mr Chris Bonnett, Africa Projects Director, GE Healthcare
Ms Sandra Lambert, Chief Commercial Officer, Biovac, South Africa.
Mr Kwasi Boahene, Director Advocacy and Programme Development, Pharmaccess

11:00–11:30

Health break and networking:
Exhibition space

11:30–12:30

Health Security in Africa: From Preparedness to Response: Collaboration for Improved Coordination, Preparedness and Global Health Security – Salão Nobre

Dr Joseph Cabore , Director, Programme Management

MODERATOR

Ms Denise Epote, Regional Director for Africa, TV5Monde

12:30–14:30

Lunch and networking

13:00–14:30

Side event 1: Cabo Verde Good Practices for Universal Health Coverage – Sala Jornadas 1

Presidential Initiative, More Life, Less Alcohol
Dr Manuel Faustino

Programme Mexi-Mexe
Dra Ivanilda Reis, Advisor to the Minister of Sports

Reduction of mortality in children under 5 years
Dr Yorleydis Rosabal & Dr Tomas Valdez

Health financing
Dr Serafina Alves & Dr Yolanda Estrela

Elimination of HIV transmission from mother to child **Dr Jorge Barreto & Dr Artur Correia**

MODERATOR

Mr Alveno Figueiredo, Media Advisor, National Assembly of Cabo Verde

13:15–14:15

Side event 2: Integrated Disease Surveillance and Response: Celebrating 20 Years of Integrated Disease Surveillance and Response in Africa – Sala Jornadas 2

PANELLISTS

Dr Ambrose Talisuna, Health Security Advisor, WHO Regional Office for Africa

Dr Hellen Perry, Retired, US Centers for Disease Control and Prevention and IDSR expert

Dr Wondimagegnehu (Wondi) Alemu,
Chief Technical Officer, International Health Consultancy and IDSR expert

Dr Thelma Nelson,
Director, Division of Global Health, Liberia

Dr Lionel Wilfrid Landaogo Ouedraogo,
Director, Health Protection, Burkina Faso

MODERATOR

Mr Henry Bonsu, International broadcaster

14:30–16:30

Session 4, Part One: Health Security: Opportunities and Challenges – Salão Nobre

Africa faces huge health challenges – logistical, financial and human – with most countries lacking good-quality and affordable health services. Millions of Africans fall into poverty due to high out-of-pocket payments, and too many are unable to access the health services that they need. This is further exacerbated by natural disasters, droughts, floods, disease outbreaks and epidemics, poor sanitation and lack of universal access to safe drinking water. With unplanned and rapidly expanding urbanization, increasing impacts from climate change and changing disease trends, public health emergencies are expected to emerge in areas unaffected previously. The continent battles against more than 100 significant public health events each year already. Universal health coverage means that even when there are disease outbreaks or disasters, preparedness and effective rapid response are in place and essential services remain available (for example, antenatal and vaccination services). Currently, many of the necessary services are interrupted and often unavailable in such circumstances. Despite existing frameworks and strategies, such as the International Health Regulations (IHR 2005), the Integrated Disease Surveillance and Response framework and the disaster risk management strategies, tackling outbreaks and other health emergencies continues to be challenging. The latter has been attributed to the fragmented implementation of interventions, limited intersectoral collaboration, inadequate resources, weak health systems and inadequate IHR 2005 capacities.

Invited expert speakers, comprising a range of African and international leaders in public health and international development from government, academia, health professions and development partners, will share their insights and solutions on how governments can deliver sustainable health services to all people everywhere all the time as well as being able to effectively manage erupting emergencies. They will analyse innovative means for delivering essential services, given the increased need in a financially and resource-constrained environment. They will also explore how to ensure that essential health services are maintained for the entire population without losing the progress that has been made with existing services.
Session 4 will emphasize how public health emergencies have the potential to threaten African and global peace and security, disrupt national economies and destroy health systems and communities. Disease outbreaks and other public health emergencies can de-humanize communities.

In the first part of Session 4, the potential impact of innovative research-based guidelines and capacity building through technology to advance health security will be analysed. The panellists will consider the opportunities proffered by partnerships and the mobilization of the private sector in increasing health care access and provision. Increased education and resources are needed for communities to better understand, prevent and respond to health emergencies or natural disasters, and this will be expounded upon by NGOs and regional players.

KEYNOTE ADDRESS

Health Security in Africa: Opportunities and Challenges, by **Mr Elhadj As Sy**, Secretary General, International Federation of Red Cross and Red Crescent Societies

PANELLISTS

Dr Ali Ahmed, Program Area Manager, WHO AFRO

Professor Cheikh Ibrahima Niang, Social scientist

Dr Farai Charasika, Senior Technical Advisor, Higherlife Foundation and philanthropist

Dr Desmond Williams, Country Director, US Centers for Disease Control and Prevention in Liberia

MODERATOR

Ms Denise Epote, Regional Director for Africa, TV5Monde

16:30–17:00

Health break and networking: Exhibition space

17:00–19:00

Side event: Cholera Prevention and Control – Salão Nobre

Cholera mostly affects poor and vulnerable populations and the burden and impact of cholera epidemics are particularly significant in sub-Saharan African countries, where case fatality rates regularly exceed the upper threshold of 1%. In 2017 and 2018, more than 250 000 cholera cases, including more than 5 345 deaths, representing a case fatality rate of more than 2%, were reported from 17 countries in the WHO African Region. To address this situation in an effective and sustained manner, country ownership and "buy-in" across various government sectors in prevention, preparedness and response is vital. To facilitate the implementation of activities to reduce the cholera burden in Member States, the WHO Regional Office for Africa developed the Regional Framework for Implementation of the WHO Renewed Strategy for Cholera Prevention and Control, 2018–2030, which sets targets and milestones to achieve in order to reduce the magnitude and number of cholera outbreaks in the region. The regional strategy focuses on using an integrated multisectoral approach for preventing and responding to cholera outbreaks and mitigating the associated risk factors. This side event will include an overview of cholera in the region and the experiences learned on cholera control in South Sudan. The panel will pinpoint the main gaps and the challenges faced by countries and partners in initiating and rolling out effective interventions for cholera control and the prospects for its elimination in Africa. They will discuss the contribution of organizations, such as the IFRC, in community-based cholera control and discuss the potential methods to mobilize financial, logistical and human resources. Highlights will be insight on innovative solutions that can be implemented by African countries to improve the engagement of high-level countries leaderships, donors and other multisectoral stakeholders for ending cholera outbreaks in the region.

PANELLISTS

Dr Matshidiso Moeti, WHO Regional Director for Africa

Dr Fatoumata Nafo-Traoré, Regional Director for Africa, International Federation of Red Cross and Red Crescent Societies

Dr Richard Lako Lino, Director of Planning, Ministry of Health, South Sudan

Dr Abel Kabalo, Director Health Promotion, Environmental and Social Determinants, Zambia

Dr Joshua Obasanya, Representative of the Director-General, Nigeria Centre for Disease Control

Dr Dominique Legros, Representative of Global Task Force on Cholera Control

MODERATOR

Ms Zeinab Badawi, International broadcaster and Chair of the Royal African Society

17:00–18:00

Day of the Cabo Verdean women:
Aerobics & health mega class at the National Assembly front avenue

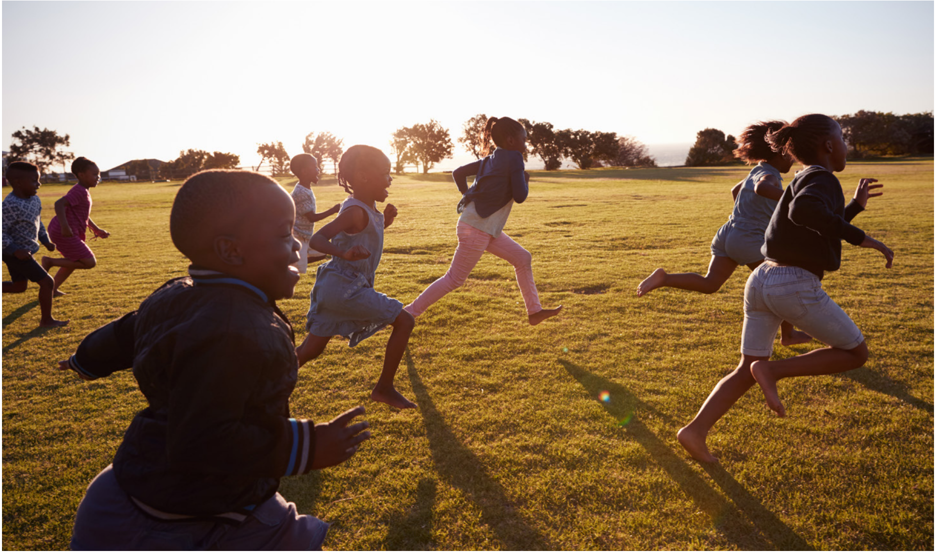

DAY THREE
28 MARCH 2019

08:00–08:55

Side event 4: Global Strategic Preparedness Network – Sala Jornadas 1

During outbreaks, the Global Outbreak Alert and Response Network (GOARN) ensures the right technical expertise and skills are on the ground where and when they are needed most. GOARN is a collaboration of existing institutions and networks, constantly alert and ready to respond. The network pools human and technical resources for rapid identification, confirmation and response to outbreaks of international importance. WHO coordinates international outbreak response using resources from GOARN. While progress has been made through GOARN on country outbreak response, country health emergency preparedness and implementation of the International Health Regulations (2005) is lagging. A global strategic preparedness network is greatly needed to help countries strengthen capacity and progress in their ability to detect, assess and respond to public health events.

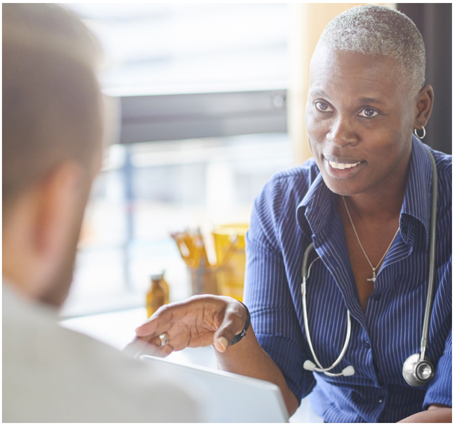

09:00–11:00

Session 4, Part Two: Health Security: An Entry Point to Universal Health Coverage – Salão Nobre

Forum participants will draw upon the panel's expertise and experience to give insight on a range of topics on universal health care and health security. Their insight will address how to galvanize partnerships; integrating innovations to build capacity; mobilizing financial, human and logistical resources; mitigating and responding to cross-border epidemics; harnessing the input of the next generation of adults; maximizing synergies to achieve universal health coverage using health security as an entry point; and engaging the media to support initiatives.

KEYNOTE ADDRESS

Contribution of Health Security to Universal Health Coverage by **Ms Hannah Brown-Amoakoh**, The Lancet Commission on Synergies, Department of Health Policy, Planning and Management, University of Ghana

PANELLISTS

Professor Francis Omaswa, Executive Director, ACHES

TMs Olamide Augustina Orekunrin, Founder, Flying Doctors Nigeria

Mr Sylvain Muzungu, CEO Tantine Group Ltd

Professor Stanley Okolo, Director General, West African Health Organization

MODERATOR

Ms Zeinab Badawi, International broadcaster and Chair of the Royal African Society

11:30–13:30

Session 4, Part Three: Sustainable Financing for Health Security in Africa – Salão Nobre

With widespread economic inequality between countries across the continent, the reality is that there are significant variations in the percentage of gross domestic product that governments allocate to health care. In addition to improving health system efficiency, there is also increasing demand for, and reliance upon, NGOs, international organizations and the private sector to remedy the financial investment gap in health care.

The panellists will deliberate on how to enlist the financial support of multilateral banks, the role of multilateral organizations in providing support mechanisms, such as the Pandemic Emergency Financing Facility, and the impacts they expect from such interventions. Consideration will be given to alternative innovative financing mechanisms that could be harnessed and the advice that they would give to African governments on how to raise the required financing for health security and universal health coverage. Sustainability will be central to the discussions, and participants will also consider the potential safeguards afforded by risk protection and insurance mechanisms for health security.

KEYNOTE ADDRESS

Domestic Financing for Sustainable Health Security in Africa by **H.E. Dr Jean Paul Adam**, Minister of Health, Seychelles

PANELLISTS

Dr Gaston Sorgho, Practice Manager, Health Nutrition and Population, Global Practice, World Bank

Dr Farai Charasika, Senior Technical Advisor, Higherlife Foundation and philanthropist

Mr Robert Kwame de Graft Agyarko, Lead Advisor, Outbreak and Epidemic, African Risk

Ms Päivi Sillanaukee, Permanent Secretary, Ministry of Social Affairs and Health, Finland and co-chair, Joint External Evaluation Alliance

Mr Babatunde Olumide Omilola, Manager, Public Health, Security and Nutrition Division, African Development Bank

MODERATOR

Dr Joannie Bewa, Public health and public policy specialist

13:30–15:30

Lunch and networking: Restaurant and tent

14:15–15:15

Side event 5: GIS technology

The WHO AFRO Polio Geographical Information Systems (AFRO Polio GIS) technology and centre was established in 2017 with funding from the Bill and Melinda Gates Foundation to support surveillance and immunization activities in the field and also monitor in real time any planned activities and supportive supervision being carried out. Additionally, because the shared data from the field is geo-coded, it allows verification and validation of submitted data, which is critical for improving data quality, particularly for certification of polio eradication. The technology is easily adaptable and versatile for any public health event. It has also been used to support interventions beyond polio, such as immunization, cholera outbreaks, meningitis outbreaks, measles outbreaks, measles campaign coverage surveys, Lassa fever outbreaks, EPI coverage surveys and mortality surveys in inaccessible and hard-to-reach areas. The technology is relatively cheap, requires very minimal investment and is sustainable. For most participants in the network, the investment is just an android mobile phone. The WHO AFRO Polio GIS technology has been rapidly expanded to tens of thousands of users in less than two years. Due to its low-cost investment, there is expectation that the technology will be sustained in Member States, beyond the polio application. The number of countries with established WHO AFRO Polio GIS technology has increased from fewer than 5 in 2017 to 43 in 2018 and the capabilities are housed in health ministries, partners and WHO country offices. Due to its user-friendliness, the technology has been expanded to community informants in hard-to-reach and inaccessible areas and areas without formal health services. As of December 2018, there were more than 5 000 community informants on the AFRO Polio GIS network.

The event will showcase the versatility and real-timeliness of the technology and the adaptability of the technology for other health interventions beyond polio, including its contribution to universal health coverage. The presentation will be followed by a plenary question and answer session.

PANELLISTS

Dr Gerald Sume , Polio Team WHO-Nigeria

Mr Godwin Akpan, Data Management Officer, WHO Regional Office for Africa

Dr Maria da Luz Lima, President, National Institute of Public Health, Cabo Verde

MODERATOR

Dr Magaran Bagayoko, Director a.i., Communicable Diseases Cluster, WHO Regional Office for Africa

15:30–17:00

Closing ceremony: Salão Nobre

MASTER OF CEREMONIES

Mr Alveno Figueiredo, Media Advisor, National Assembly of Cabo Verde

H. E. Dr Arlindo Nascimento do Rosário, Minister of Health and Social Security, Cabo Verde

Dr Matshidiso Moeti, WHO Regional Director for Africa

H.E. José Ulisses de Pina Correia e Silva, Prime Minister of Cabo Verde

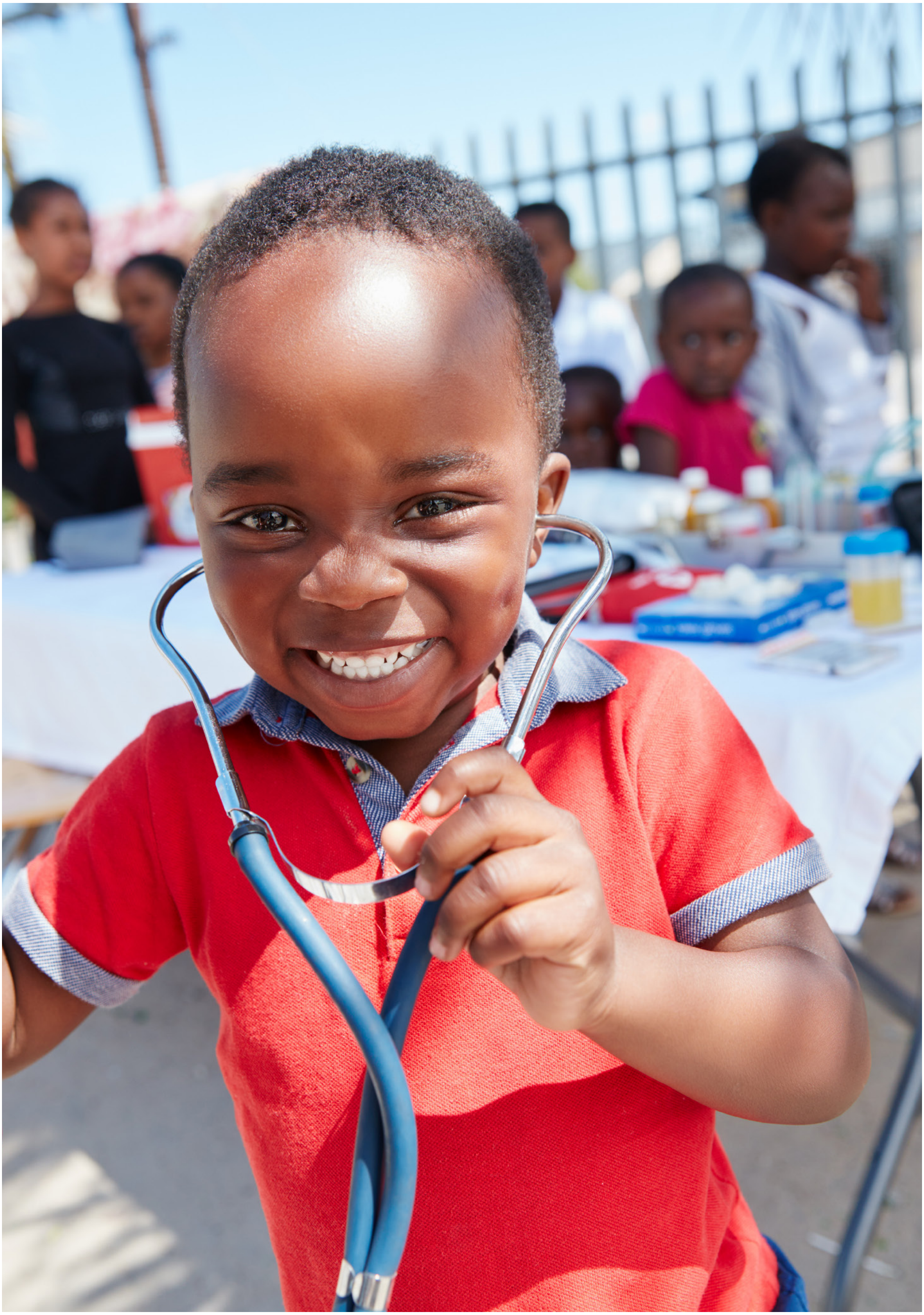

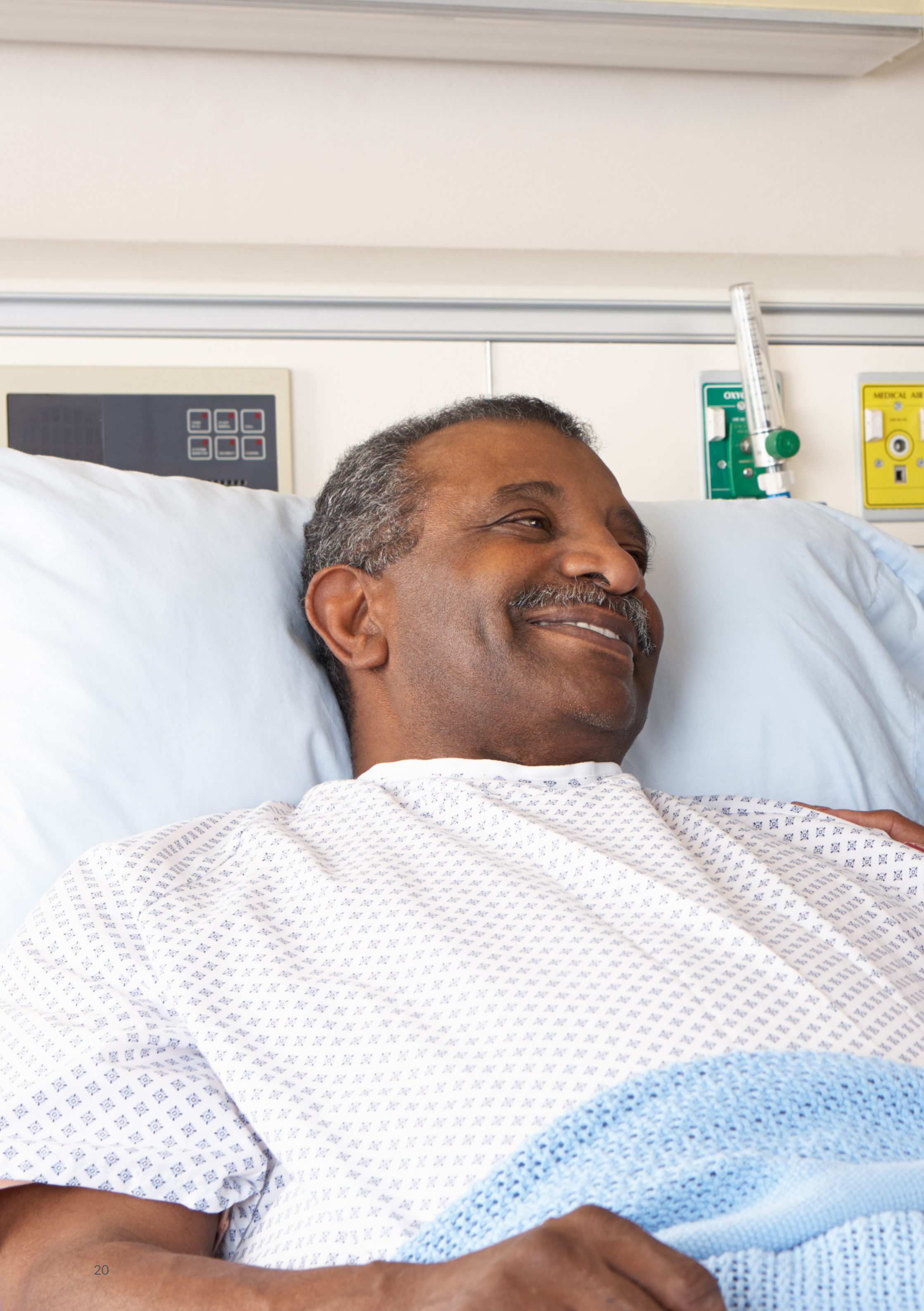

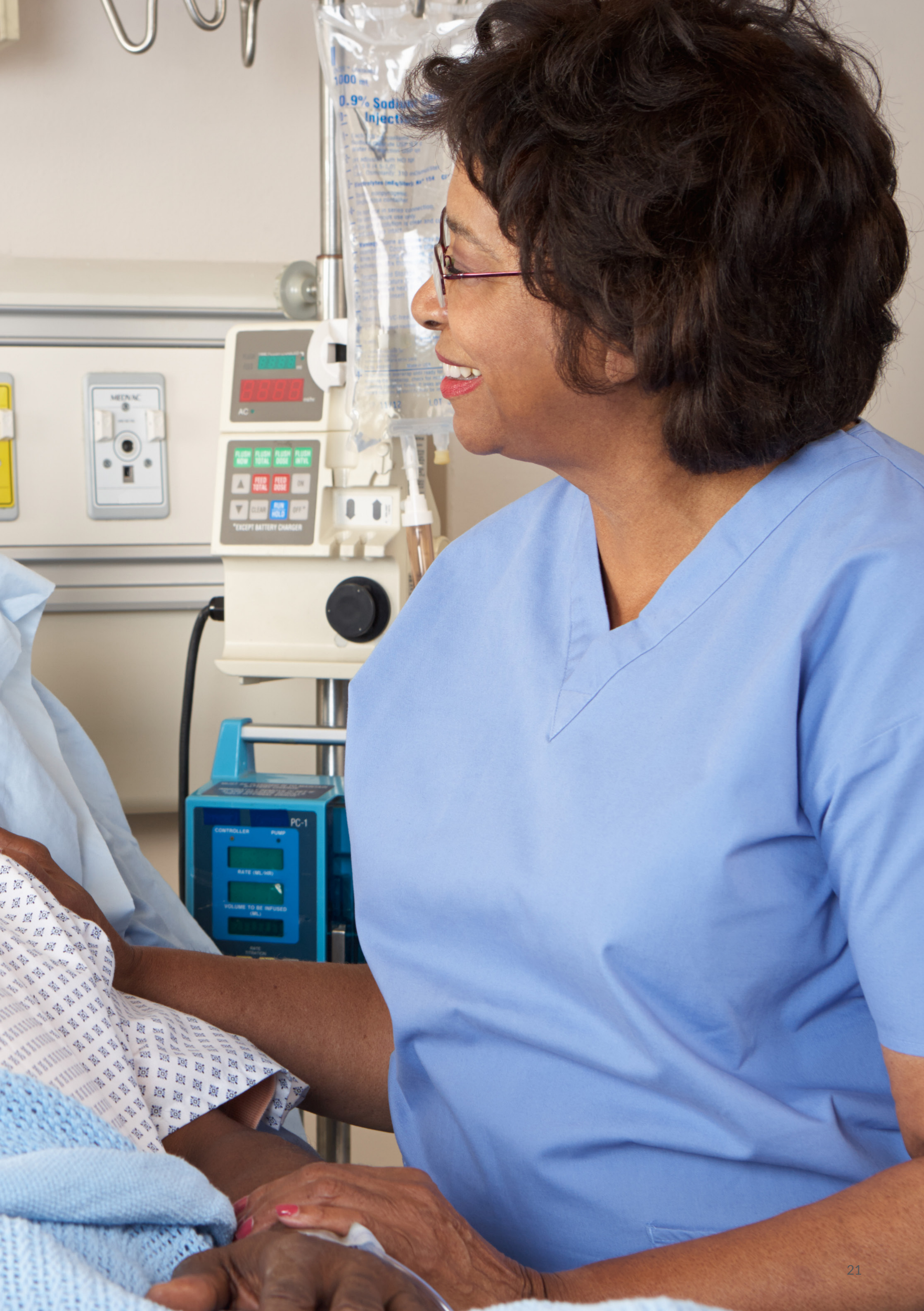
PC-1
CONTROLLER
PUMP
*EXCEPT BATTERY CHARGER

WHO SENIOR MANAGEMENT IN THE AFRICAN REGION

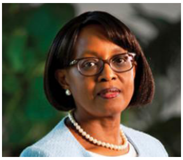

DR MATSHIDISO MOETI
WHO REGIONAL DIRECTOR FOR AFRICA
MOETIM@WHO.INT

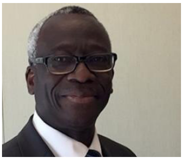

DR JOSEPH CABORÉ
DIRECTOR, PROGRAMME MANAGEMENT
CABOREJ@WHO.INT

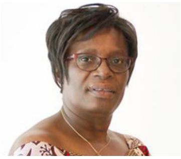

DR FELICITAS ZAWAIRA
DIRECTOR, FAMILY AND REPRODUCTIVE HEALTH CLUSTER
ZAWAIRAF@WHO.INT

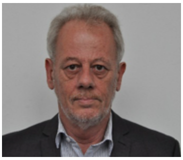

ALAN RITCHIE
DIRECTOR, GENERAL MANAGEMENT (a.i.)
RITCHIEAL@WHO.INT

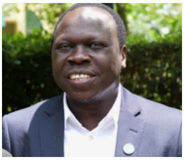

DR ZABULON YOTI
DIRECTOR, REGIONAL EMERGENCIES (a.i.)
YOTIZA@WHO.INT

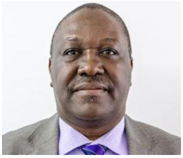

DR FRANCIS CHISAKA KASOLO
DIRECTOR, OFFICE OF THE REGIONAL DIRECTOR (a.i.)
KASOLOF@WHO.INT

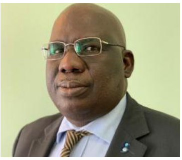

DR MAGARAN BAGAYOKO
DIRECTOR, COMMUNICABLE DISEASES CLUSTER (a.i.)
BAGAYOKOM@WHO.INT

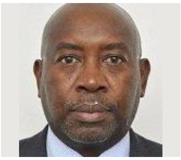

DR PROSPER TUMUSIIME
DIRECTOR, HEALTH SYSTEMS STRENGTHENING CLUSTER (a.i.)
TUMUSIIMEP@WHO.INT

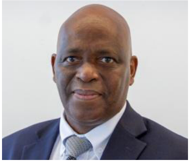

DR STEVEN V. SHONGWE
DIRECTOR NON-COMMUNICABLE DISEASES CLUSTER (a.i.)
SHONGWES@WHO.INT

ABOUT WHO | AFRICAN REGION

The World Health Organization contributes to a better future for people everywhere. Good health lays the foundation for vibrant and productive communities, stronger economies, safer nations and a better world. As the lead health authority within the United Nations system, our work touches people's lives around the world every day. In Africa, WHO serves 47 Member States and works with development partners to improve the health and well-being of all people living here. The WHO Regional Office for Africa is located in Brazzaville, Republic of Congo. Learn more at www.afro.who.int and follow us on Twitter, Facebook and YouTube.

ABOUT CABO VERDE

Cabo Verde is a 10-island nation located in the middle of the Atlantic Ocean, one hour away by plane from mainland Africa, three hours from Brazil and four hours from continental Europe. The islands of Cabo Verde, volcanic in origin, are blessed with mild temperatures all through the year and a dry tropical climate that characterizes their landscape. The archipelago's population is around half a million, with a large diaspora of more than 1.2 million people. Its capital city, Praia, is located in its largest island: Santiago. Residents and visitors enjoy its Creole culture, historic towns, black and white sandy beaches, unspoiled nature, mountains and volcanoes, water sports, great cuisine and its world-famous music that reflects strong cultural ties with the African mainland and Europe as well as with Brazil and the Caribbean islands. Cabo Verde is a republic, with President Jorge Carlos Almeida Fonseca its Head of State since 2011. José Ulisses Correia e Silva, who leads the Movement for Democracy party, became Cabo Verde's Prime Minister in 2016.

THE 2ND
WHO AFRICA
HEALTH FORUM

World Health Organization
Regional Office for Africa
Cité du Djoué
P.O. Box 06
Brazzaville
Republic of Congo
0047 241 39100

Tel: + (242) 06 508 11 14
Fax: + (47 241) 39503

Email: AfricaHealthForum@who.int
www.afro.who.int

World Health Organization
REGIONAL OFFICE FOR Africa
