# Supplementary material for: Community participation and private sector engagement are fundamental to achieving universal health coverage and health security in Africa: reflections from the second Africa health forum
Source: BMC Proc. 2019 Nov 12;13(Suppl 9):7. doi: 10.1186/s12919-019-0170-0 (PMC6849158; doi:10.1186/s12919-019-0170-0)
Supplement: Supplementary file 2 — Additional file 2. Communique of the second WHO Africa Health Forum. [file 12919_2019_170_MOESM2_ESM.pdf]

World Health Organization
REGIONAL OFFICE FOR Africa

MINISTÉRIO DA SAÚDE E DA SEGURANÇA SOCIAL

GOVERNO DE CABO VERDE
A TRABALHAR PARA TODOS.

Second WHO Africa Health Forum

Praia, Cabo Verde

COMMUNIQUE

The Second World Health Organization Africa Health Forum (WAHF) was jointly convened by the Government of Cabo Verde and the World Health Organization Regional Office for Africa in Praia, Republic of Cabo Verde from 26 to 28 March 2019. It was a follow-up to the First WAHF organized in June 2017 in Kigali, Rwanda which committed to *"putting people first, promoting synergies and coordination and engaging all stakeholders behind the goal of achieving universal health coverage while leaving no one behind"*.
Organized under the distinguished patronage of His Excellency E. Jorge Carlos Almeida Fonseca, President of the Republic of Cabo Verde and His Excellency Jose Ulisses Correira e Silva, Prime Minister of the Republic of Cabo Verde, the Forum was attended by a broad and diverse range of participants, including high-ranking government officials from the ministry of health and other sector ministries, parliamentarians, civil society organizations and youth representatives, the media, agencies of the United Nations system and other stakeholders.

The theme for the Forum was **"achieving universal health coverage and health security: the Africa we want to see."** The theme underscored the central role of good health and the importance of ensuring health security and universal health coverage for the continent's sustainable development. The Forum also highlighted the need for strategic partnerships, effective engagement and coordinated actions for better management and mitigation of the urgent and ever-changing health needs of African populations.

The key thematic areas for deliberation during the Forum were aimed at taking UHC to the next level; optimizing multisectoral partnerships for effective collaboration to improve health outcomes; ensuring health security; and promoting innovations for the achievement of UHC.

The following are the main recommendations of the Forum.

On the need to take UHC to the next level:

Member States should:

- scale-up implementation of the universal health coverage strategy as an effective way to guarantee the right to health for all without major financial constraints, thereby contributing to the social well-being of their populations and to the achievement of the Sustainable Development Goals;

- accelerate the strengthening of national health systems, focusing on the primary health care strategy as the preferred pathway to achieving universal health coverage, as reiterated in the Declaration of Astana on Primary Health Care;
- ensure active community participation in deciding, implementing and monitoring the effects of health initiatives;
- mobilize additional funding and improve the quality and efficiency of investments to accelerate and scale up efforts towards UHC.

Governments and Partners should:

- actively monitor the range of essential health services available to each age group in countries to ensure that citizens enjoy greater access to the services they need for their health and well-being;
- prioritize initiatives focusing on communities, facilities and districts to build the resilience of health systems, to ensure sustained provision of essential services;
- support the expansion of health promotion, disease prevention, curative, rehabilitative and palliative interventions, particularly for the populations currently left behind;
- proactively support the generation and use of the data and statistics needed to monitor progress towards UHC in Member States, and make adjustments when necessary;
- move away from payment at the point of use as it is associated with inequities and financial barriers to access to services.

On the need to optimize multisectoral partnerships for effective collaboration to improve health outcomes:

Member States should:

- promote intercountry stakeholders' dialogue and public–private partnerships including with intergovernmental organizations, the private sector, development banks and regional economic communities, nongovernmental organizations, local authorities, research institutions and academia;
- strengthen public-private partnerships for health and well-being at all levels of government and across key partner agencies to enhance health promotion, prevention and care policies and actions;
- engage with and ensure the meaningful participation of the citizenry, including the youth, women and other vulnerable groups in the development and implementation of policies and strategies affecting their health and well-being;
- support local government and community structures to address the determinants of health and health inequities to ensure that no one is being left behind;
- invest in gathering and using the strategic information needed for advocacy, planning and monitoring of programmes for adolescent and youth health;
- work to reduce policy barriers that limit access for young people and other vulnerable groups to health information and services (for example, HIV testing and contraception) and accelerate the development and implementation of policies that protect young people and promote their health and well-being.

Governments and Partners should:

- continue to promote "Health in All Policies" and support cross-sectoral and intersectoral policy coherence and actions in order to address the determinants of health and to improve the health and well-being of the population;
- empower the health sector to effectively engage and support the other sectors to incorporate "Health in all Policies" in their processes;

WHO and other agencies of the UN system should:

- work with Member States, development partners and financial institutions to harmonize and integrate policies, strategies and high-impact interventions while expanding efforts to reach the most vulnerable populations and improving equity;
- support Member States in mobilizing funding and improve the quality and efficiency of investments to strengthen multisectoral and community engagement and; scale up innovative new tools and approaches;
- engage Heads of State and Government in championing a systematic and coherent multisectoral agenda for addressing the key determinants of health in their countries;
- support private and public sector investments in health promotion and primary prevention;
- provide evidence-based guidance that supports healthy choices and interventions, while applying the WHO Framework of Engagement with Non-State Actors.

On the need to enhance private sector engagement for UHC and health security through evidence-based actions:

Member States should:

- put in place an enabling legal and policy environment and instruments that regulate the engagement and role of the private sector in UHC and health security;
- create and institutionalize forums for ongoing dialogue between health sector partners and the private sector;
- identify suitable areas for engaging and contracting the private sector to expand service coverage;
- develop adequate accountability frameworks to ensure mutual transparency and accountability *vis-à-vis* the private sector.

The Private Sector should:

- commit to working with governments to achieve the social contract that is inherent in UHC within mutually agreed accountability mechanisms and frameworks;
- leverage existing resources (technical, infrastructure, ICT) to provide innovative solutions for progress towards UHC and health security in Africa in order to ensure that no one is left behind.

WHO, other agencies of the UN system and Partners should:

- provide technical support to countries to enable them design and establish an enabling legal and policy environment as well as accountability frameworks;

- support the generation of evidence on good practices for public-private engagement to advance progress towards UHC;
- support capacity building and experience sharing among Member States to ensure that countries are well prepared to effectively harness the benefits of private sector engagement in health.

On the need to ensure health security:

Member States, WHO and other agencies of the UN system and Partners should:

- accelerate full implementation of the International Health Regulations (IHR 2005) and strengthen cross-border collaboration;
- propose a mechanism with clear, actionable next steps for improving collaboration and coordination for public health emergency preparedness, response and global health security at national, regional and global level, while paying attention to the "One Health" approach;
- define the roles and responsibilities of the different stakeholders (WHO, the Africa CDC, international and national NGOs, international agencies, bilateral and multilateral funders, academia and researchers) in supporting countries to fast-track the achievement of health security;
- agree on an integrated mechanism with clear timeliness for formulating a resource mobilization strategy for sustainable (domestic and external) financing to support the implementation of the National Action Plan for Health Security (NAPHS) using a phased and focused approach among and within countries, and ensure its linkage with sector plans;
- conduct research to improve the epidemiological knowledge and risk factors of, and enhance response interventions to, the top five major causes of infectious disease outbreaks in Africa.

On the need to promote innovations for the achievement of UHC and health security:

Member States should:

- provide fiscal and non-fiscal incentives to support the development of health innovations;
- support the development and implementation of innovation-friendly policies;
- develop effective strategies for incorporating innovations and new technologies into health sector interventions;
- institutionalize the use of GIS technological innovations to monitor and accelerate progress towards universal health coverage, including preparing for and responding to health emergencies.

WHO, Partners and the Private Sector should:

- play a leading role in harnessing and supporting the scale-up of health innovations from the African continent;
- identify, document and share good practices across countries, including lessons learned from interregional, South-South and triangular cooperation, in order to foster a culture of innovation internally and externally;
- scale up the promotion of technological integration and innovation in health among Member States, including showcasing exhibitions on innovations during high profile meetings and conferences.

The Forum also recommended that Member States, Partners and Donors accelerate implementation of the "Kigali Call to Action" to significantly contribute to the achievement of the "triple billion" targets of the WHO Thirteenth General Programme of Work as adopted by the Seventy-first World Health Assembly.

During the closing ceremony, the Forum extended its sincere gratitude to His Excellency E. Jorge Carlos Almeida Fonseca, President of the Republic, His Excellency Jose Ulisses Correira e Silva, Prime Minster, and the Government and People of Cabo Verde for successfully hosting the Second Africa Health Forum.

The Forum requested the WHO Regional Director for Africa to present this "Communique" to the Sixty-ninth session of the WHO Regional Committee for Africa.

In line with the decision taken during the First WAHF to hold the Forum every two years, the next Forum will be held in 2021 at a venue to be determined.

Issued by:

Dr Matshidiso Moeti<br>WHO Regional Director for Africa	Dr Arlindo Nascimento do Rosário<br>Minister of Health and Social Security,<br>Cabo Verde
28 March 2019	28 March 2019
